# WHO, ME, WORRIED?: A LOOK AT BRAIN HEALTH AND STRESS AMONG RESIDENTS IN GEOGRAPHICALLY DIFFERENT AREAS

**DOI:** 10.1093/geroni/igz038.1299

**Published:** 2019-11-08

**Authors:** Katherine Bridges

**Affiliations:** AARP, Washington, District of Columbia, United States

## Abstract

The 2018 AARP Brain Health and Mental Well-Being Survey reveals Millennials (age 22 to 37) have the highest level of stress while those in the Silent/Greatest Generation (over 73) have the lowest. Adults in their 50s and beyond have higher average mental well-being scores compared to younger adults. On a scale of mental well-being with an average score of 52, the average well-being for those age 18-39 is about 50, compared to about 54 for those 60 and older. This presentation will highlight generational difference in mental well-being and will examine community difference for older adults particularly those who reside in metropolitan areas compared to non-metropolitan areas.

